# A Comparison of the Efficacy and Safety of US-, CT-, and MR-Guided Radiofrequency and Microwave Ablation for HCC: A Systematic Review and Network Meta-Analysis

**DOI:** 10.3390/cancers17030409

**Published:** 2025-01-26

**Authors:** Hao Li, Thomas J. Vogl, Kuei-An Chen, Hamzah Adwan

**Affiliations:** 1 Clinic for Radiology and Nuclear Medicine, University Hospital Frankfurt, Johann Wolfgang Goethe University, Theodor-Stern-Kai 7, 60590 Frankfurt, Germany; haolimed90@gmail.com (H.L.); adwan.hamza97@gmail.com (H.A.); 2 Department of Medical Imaging and Intervention, Chang Gung Memorial Hospital, Chang Gung University, Linkou, Taoyuan City 33305, Taiwan; chenkueian@gmail.com

**Keywords:** hepatocellular carcinoma, magnetic resonance, computed tomography, ultrasound, thermal ablation

## Abstract

**Objectives:** The aim of this study was to compare the efficacy and safety of thermal ablation, focusing on radiofrequency ablation (RFA) and microwave ablation (MWA), for hepatocellular carcinoma (HCC) using US-, CT-, and MR-guidance. **Methods:** PubMed, EMBASE, Cochrane Library, and Web of Science were searched for studies comparing US, CT, and MR guidance in thermal ablation for HCC. Observational studies and randomized controlled trials (RCTs) were included. Overall survival (OS), local tumor recurrence (LTR), primary technique effectiveness (PTE), and major complications were assessed with network meta-analysis. **Results:** One RCT and 13 retrospective cohort studies reporting on 2349 patients were included. For OS at 3 years, compared to CT, US had hazard ratios (HRs) of 0.98 (95%CI: 0.77–1.26), and MR had HRs of 1.60 (95%CI: 0.51–5.00); For OS at 5 years, US had HRs of 0.80 (95%CI: 0.64–1.01), and MR had HRs of 1.23 (95%CI: 0.52–2.95) compared to CT. LTR rates, PTE, and major complications did not show statistical significance among the three guidance modalities (LTR: RR = 0.29 (95%CI: 0.08–1.14), *p* = 0.97 MR vs. CT; RR = 0.25 (95%CI: 0.06–1.02), *p* = 0.97 MR vs. US; PTE: RR = 1.06 (95%CI: 0.96–1.17), *p* = 0.90 MR vs. CT; RR = 1.08 (95%CI: 0.98–1.20), *p* = 0.90 MR vs. US. Major complications: RR = 0.27 (95%CI: 0.13–0.59), *p* = 0.94 MR vs. CT; RR = 0.41 (95%CI: 0.10–1.74), *p* = 0.94 MR vs. US). **Conclusions:** CT-, US-, and MR-guided RFA and MWA are equally effective and safe for HCC patients.

## Simple Summary

Thermal ablation is a common treatment for liver cancer (HCC) performed under the guidance of various imaging techniques, including ultrasound, computed tomography, and magnetic resonance imaging. This study compared the effectiveness and safety of these three guidance methods by analyzing data from 2349 patients across multiple studies. The results indicated that all three techniques were similarly effective and safe for HCC treatment. While MR guidance showed some advantages in specific outcomes, these findings did not reach statistical significance. Therefore, all three imaging techniques remain reliable options for guiding thermal ablation in HCC patients.

## 1. Introduction

Liver cancer is a significant contributor to the global cancer burden, with incidence rates rising in many countries in recent years. Hepatocellular carcinoma (HCC) is the primary histologic type of liver cancer, responsible for most liver cancer cases and fatalities [1]. The optimal clinical decision for HCC is a multifaceted issue that necessitates the careful consideration of tumor characteristics (size, number, and vascular invasion), liver status (Child-Pugh score), and functional status of the patients [2,3]. Currently, treatments for HCC mainly include surgical resection, liver transplantation, transarterial chemoembolization, and image-guided thermal ablation, among others. However, some patients may not be suitable for the radical treatment options due to various factors such as high surgical costs, limited availability of liver sources, or underlying diseases. As a result, minimal-invasive treatments have been increasingly used as an effective alternative treatment for HCC, among which thermal ablation is generally accepted by most clinical centers [4,5,6].

Commonly used ablation techniques of HCC, such as radiofrequency ablation (RFA) and microwave ablation (MWA), primarily rely on inducing thermal changes in the targeted area. While RFA has been extensively researched, MWA is gaining popularity due to its ability to create a larger ablation zone in a shorter time [7]. However, the application of thermal ablation cannot be separated from the image guidance, with ultrasound (US), computed tomography (CT), and magnetic resonance (MR) being the typical imaging modalities. Moreover, the choice of image guidance modality significantly influences the safety and effectiveness of thermal ablation [8,9].

During the guidance of the ablation procedure, each imaging technique has its own advantages and disadvantages. Compared to CT and MR, US has the most significant advantage of real-time imaging, which allows the operator to observe the depth and direction of the needle in real time and insert the needle into the target tumor under real-time monitoring. In addition, US is user-friendly, radiation-free, cost-effective, and provides a clear visualization of blood vessels and the bile duct. However, US does have some drawbacks including low image resolution; limited location accuracy; interference from nearby organs such as the ribs, lungs, and gastrointestinal tract; and challenges in evaluating the extent of ablation [10]. In contrast, CT can largely avoid these drawbacks [11,12]. However, non-enhanced CT cannot clearly define the boundaries of the ablation lesions, and contrast-enhanced CT, which has a need for the repeated use of contrast media, undoubtedly increases the burden on the kidneys. Furthermore, with the increasing number of scans performed, concerns regarding exposure to ionizing radiation, which may lead to various degrees of impairment to interventionists and patients, have also increased [13]. MR imaging offers many potential advantages that make it a promising tool to guide thermal ablation. These advantages include being radiation-free, providing high soft tissue contrast, and three-dimensional scanning [14,15]. More importantly, MR allows for live thermometry to monitor thermal distribution and deposition in real time, which helps reduce the incidence of normal tissue damage around the targeted lesion. However, factors such as the high cost of MR scanners, complex sequences, and the absence of MR-compatible ablation applicators limit its clinical use.

While various image-guided thermal ablation treatments for HCC have been commonly utilized in clinical practice, there remains a lack of consensus regarding the equivalency of US, CT, and MR guidance. Thus, we aimed to perform a network meta-analysis to evaluate the relative efficacy and safety of CT-, US- and MR-guided thermal ablation for the treatment of HCC.

## 2. Materials and Methods

### 2.1. Protocol and Registration

The study was reported according to the PRISMA-NMA extension statements for network meta-analysis [16] (Supplementary Table S8). The protocol has been registered with PROSPERO (registration number: CRD 42023423751).

### 2.2. Eligibility Criteria

#### 2.2.1. Studies

Randomized controlled trials (RCTs) and observational studies comparing two or three guidance modalities of thermal ablation for the treatment of HCC, irrespective of the use of allocation concealment or blinding, with complete patient information.

#### 2.2.2. Participants

All patients diagnosed with HCC undergoing treatment with ultrasound-, CT-, or MR-guided thermal ablation were included. Patient eligibility was not restricted by age, race, or disease duration, provided they met the treatment indications for image-guided thermal ablation and had no apparent contraindications.

#### 2.2.3. Interventions

Studies that compared the guidance modality for thermal ablation among CT, US, and MR, or compared two out of these three modalities.

#### 2.2.4. Outcomes

Considering that the majority of the included population comprised patients with HCC, the main aim was to address concerns regarding survival and local tumor control. Based on this, comparative studies that reported outcomes related to survival and local tumor control were eligible.

#### 2.2.5. Excluded Criteria

The studies comparing image-guided thermal ablation with laparoscopic or open surgical approaches for thermal ablation were excluded. The fusion imaging-guided thermal ablation utilizing multiple imaging modalities was excluded. Although studies comparing thermal ablation guided by CT, US, and MR were conducted, those lacking results relevant to this study were excluded. Duplicate publications, including multiple abstracts or papers authored by the same team, were excluded from the analysis.

### 2.3. Search Strategy

This study searched the PubMed, Embase, Web of Science, and Cochrane Library databases to find all the relevant published literature until 31 May 2023 using the terms included in Supplementary Table S1. The search focused on identifying the literature concerning the effectiveness and safety of thermal ablation under CT, US, and MR guidance. The retrieved literature’s reference lists underwent manual scrutiny to uncover further potentially relevant studies.

### 2.4. Study Selection

Conforming to the standard methodology of network meta-analysis, we exclusively included studies with comparative designs, guaranteeing a minimum of two arms in each study. The process of selecting studies included 2 levels and a qualitative assessment. Initially, two researchers independently reviewed and managed the literature using the EndNote X9 software. Duplicate publications were identified and eliminated through software deduplication and manual verification. The remaining literature underwent screening according to the predetermined inclusion and exclusion criteria. Level 1: the articles underwent title and abstract screening to identify those that presented comparisons between at least two image-guided modalities in a comparative study (i.e., cohort study, or RCT); Level 2: the selected articles underwent full-text screening to filter out studies lacking relevant outcome indicators or not strictly adhering to the intervention measures specified in the criteria. In the end, the included literature was cross-checked by two researchers, and any disagreements were resolved through discussion or consultation with a third reviewer.

### 2.5. Data Extraction

Two reviewers extracted the data using an electronic form independently. Any disagreements should be negotiated and agreed upon with the third reviewer. The data primarily involved study demographic information and outcome indicators. The demographic information included general information such as the author’s name, year of publication, sample size, age of the cases, tumor size, etc. And, the detailed data of the outcome indicators were listed as follows:

1. Overall survival (OS) at 3 years and at 5 years;
2. Local tumor recurrence (LTR): LTR was defined as the appearance of any new tumor foci at the edge of an ablation zone, detected after at least one dynamic follow-up study confirming adequate ablation;
3. Primary technique effectiveness (PTE): PTE was defined as the complete ablation of the target tumor on the first follow-up image;
4. Major complications: A major complication was defined as an event that causes significant morbidity and disability, necessitates an increased level of care, leads to hospital admission, or significantly prolongs the hospital stay (Society of Interventional Radiology (SIR), classifications C–E).

Hazard ratios (HRs) and their standard error (SE) were used to evaluate the 3-year and 5-year OS, which was extracted or calculated as follows:

1. If the report includes both unadjusted and adjusted statistics, we opted to extract the adjusted data. In the case of multi-arm trials, we conducted the analysis by computing the standard error (SE) of the control group using the formula outlined by Woods et al. [17].
2. If the HR was not directly provided but a survival curve with an at-risk table was available in the article, we calculated the HR and its 95% confidence interval (CI) using the electronic computing table developed by Tierney et al. [18].

The LTR, PTE, and major complications used dichotomous outcomes. Relative risk (RR) was used to evaluate LTR, PTE, and major complications, respectively.

### 2.6. Assessment of Risk of Bias

The included studies underwent rigorous risk-of-bias assessment using tailored scales specific to their study types. Two researchers independently evaluated various scales. Any discrepancies were resolved through discussion with the third reviewer.

The revised Cochrane risk-of-bias tool (ROB 2.0) [19] was used for the evaluation of the risk of bias in the randomized trials. The assessment criteria included evaluating bias stemming from randomization, deviations from intended intervention, missing data, outcome measurement, and the selection of reported results. Each individual aspect was assessed as either “Low”, “High”, or “Some concerns”. If all the aspects were rated as low risk, the study received a “Low” rating. Conversely, if any aspect was deemed high risk, the study was rated as “High”. Otherwise, if there were concerns in some aspects, the rating was categorized as “Some concerns”.

The Newcastle–Ottawa scale [20] was first used to preliminarily identify the quality of the retrospective studies. To comprehensively assess the bias in non-randomized studies, we further utilized the ROBINS-I tool [21] for evaluation. The evaluation criteria were structured as follows: pre-intervention domains (bias related to confounding, bias in the selection of participants into the study); at-intervention domains (bias in the classification of interventions); post-intervention domains (bias due to deviations from the intended interventions, bias due to missing data, bias in the measurement of outcomes, and bias in the selection of the reported result). Every bias domain was assessed and categorized as “Low”, “Moderate”, “Serious”, or “Critical”. Based on the signaling questions and the table for reaching the risk of bias judgments in ROBINS-I, a bias risk assessment was conducted for each non-randomized study.

### 2.7. Statistical Analysis

The frequentist approach was used for conducting the network meta-analysis [22]. For survival outcomes, including 3-year OS and 5-year OS, the HR and its 95% CI were calculated. For dichotomous outcomes (LTR, PTE, and major complications), the analysis generated a Risk Ratio (RR) with 95% CI. All the data analyses were performed using the R package (version 4.3.2) *Netmeta*. The *I*^2^ statistic was used to assess heterogeneity, with values >50% indicating significant heterogeneity [23]. A random effects model was chosen due to the presumed heterogeneity among the studies. “Node splitting” was performed to detect differences between direct and indirect evidence within a closed loop (inconsistency). A *p*-value of <0.05 was considered statistically significant. The surface under the cumulative ranking curve (SUCRA) was used to calculate the probabilities of each imaging modality being the best among all the options.

## 3. Results

### 3.1. Literature Search Results

**Figure 1:**
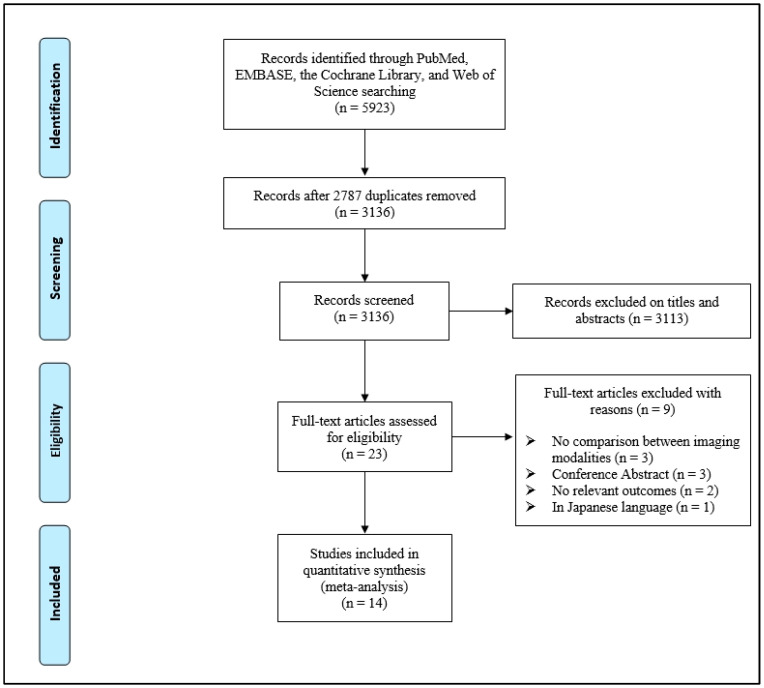
PRISMA flow diagram of the process of screening and selecting studies. PRISMA, Preferred Reporting Items for Systematic Reviews and Meta-Analyses.

From the systematic search of databases, a total of 5923 articles were initially identified. Following the removal of duplicate studies, two reviewers evaluated the titles and abstracts (level 1) of potentially eligible studies from the remaining pool of 3136 articles. During the title and abstract screening phase, 3113 records that were not in line with our research objectives were excluded. These exclusions included articles on unrelated topics, those that did not meet our inclusion criteria, and reviews or meta-analyses. We conducted a thorough eligibility assessment of the remaining 23 articles (level 2). Out of these articles, 14 articles were included for the analysis [8,24,25,26,27,28,29,30,31,32,33,34,35,36] and 9 articles were excluded, with detailed exclusion reasons presented in Supplementary Table S2. Figure 1 shows the flow diagram of the search strategy conducted in this network meta-analysis.

### 3.2. Included Study Characteristics

The key features of the included studies are outlined in Table 1. The recruitment period ranged from 2014 to 2022. One study was an RCT [29] and 13 were retrospective studies [8,24,25,26,27,28,30,31,32,33,34,35,36]. The studies included predominantly took place in Asian regions [24,25,26,29,30,31,32,33,34,35,36]. In total, 2349 participants were aggregated for the meta-analysis. Of these, 954 participants were under the CT guidance, 920 participants were under the US guidance, and 475 participants were under the MR guidance. The average tumor size ranged from 1.11 to 4.12 cm, with two studies reporting the tumor size as a median [26,30], while three studies did not provide the mean or median tumor size [24,28,32]. There were three three-arm trials [25,30,36], of which one three-arm trial was selected with only two arms for assessment [36]. For two-arm trials, nine studies [24,26,27,28,29,31,33,34,35] directly compared the CT and US guidance modalities, and two studies [8,32] compared the CT and MR guidance modalities. Between the MR and US guidance modalities, there was no direct comparison in two-arm trails. All the clinical endpoints reported in the included studies are presented in Table 2.

### 3.3. Risk of Bias

The revised Cochrane risk-of-bias tool (ROB 2.0) was used to assess the RCT study (Liu et al. [29]), which is an open-label study with an overall risk of bias categorized as “Some concerns”.

In the Non-RCT and retrospective studies, we preliminary conducted the qualitative evaluation using the Newcastle–Ottawa Scale (NOS). Twelve observation studies were considered high quality, while one study [28] was deemed as moderate quality (Supplementary Table S3). Then, we further conducted the Robins-I tool for a comprehensive evaluation: the study by Lin et al. [25] was judged to be “Critical risk”; the studies by Wu et al. [24], Si et al. [31], Li et al. [32], and Zhao et al. [36] were judged to be “Low risk”; and the others were assessed as “Moderate risk” or “Serious risk”. The detailed results are provided in Supplementary Table S4 and Supplementary Figure S1.

### 3.4. Network Geometry

**Figure 2:**
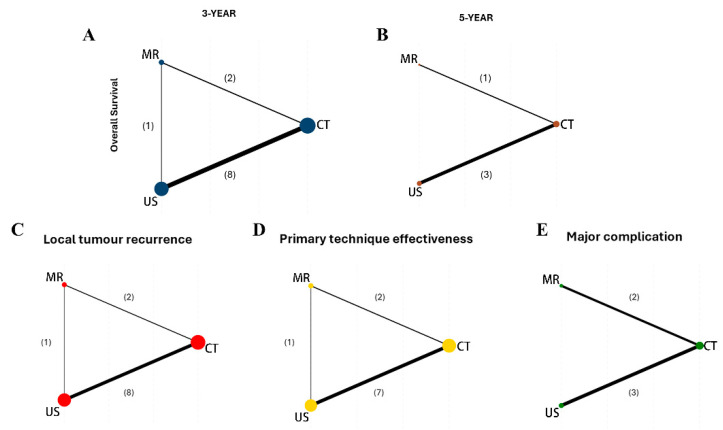
Network plots for (**A**) 3-year OS, (**B**) 5-year OS, (**C**) LTR, (**D**) PTE, and (**E**) major complications. The node (circle) size represents the number of participants, while the thickness of the connecting lines is proportional to the number of studies. The number of studies is indicated in parentheses. CT, computed tomography; MR, magnetic resonance; US, ultrasound.

A network map of intervention relationships and comparisons is presented in Figure 2. Node size and line thickness are proportional to the number of included patients and number of trials, respectively. The 3-year OS, LTR, and PTE showed a closed triangle, while the 5-year OS and major complications lacked a direct comparison between MR and US, resulting in an incomplete triangle loop.

### 3.5. Network Meta-Analysis

#### 3.5.1. OS

**Figure 3:**
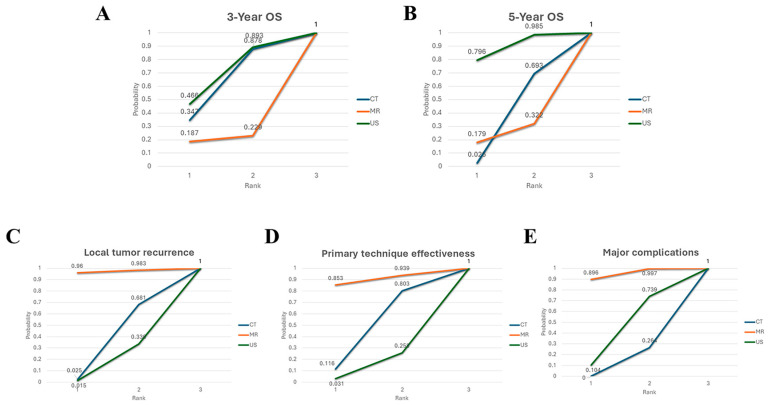
Surface under the cumulative ranking curve indicates the probability of each imaging modality being the best among the options, and the numbers in the graph represent the cumulative probabilities for each rank. (**A**) For 3-year OS, US has the highest probability of being ranked first. (**B**) For 5-year OS, US has the highest probability of being ranked first. (**C**) For local tumor recurrence, MR has the highest probability of being the best choice. (**D**) For primary technique effectiveness, MR has the highest probability of being the best imaging modality. (**E**) For major complications, MR has the highest probability of being the safest modality. CT, computed tomography; MR, magnetic resonance; US, ultrasound.

The 3-year OS was evaluated in nine studies and data on the 5-year OS was available in four studies (Supplementary Table S5). NMA included all these studies. For the OS at 3 years, as compared to CT, US had an HR of 0.98 (95%CI: 0.77–1.26), and MR had an HR of 1.60 (95%CI: 0.51–5.00); for OS at 5 years, as compared to CT, US had an HR of 0.80 (95%CI: 0.64–1.01), and MR had an HR of 1.23 (95%CI: 0.52–2.95). Overall, none of the comparisons was statistically significant. The SUCRA analysis provided a ranking of these three image modalities (Figure 3A,B). For OS at 3 years, US had the highest probability of being ranked first (SUCRA = 68), followed by CT (SUCRA = 61.3) and MR (SUCRA = 20.5). Similarly, for OS at 5 years, US again had the highest probability of being ranked first (SUCRA = 90), followed by CT (SUCRA = 36.9) and MR (SUCRA = 23.1).

#### 3.5.2. LTR

The NMA included nine studies that reported rates of LTR (Supplementary Table S6). The LTR rates were without statistically significant difference (RR = 0.29 (95%CI: 0.08–1.14), *p* = 0.97 MR compared to CT; RR = 0.25 (95%CI: 0.06–1.02), *p* = 0.97 MR compared to US). A ranking analysis based on the SUCRA scores indicated that MR (SUCRA = 96.8) has the greatest likelihood of being the best choice in terms of reducing the rate of LTR, followed by CT (SUCRA = 35.8) and US (SUCRA = 17.4) (Figure 3C).

#### 3.5.3. PTE

The NMA included eight studies for PTE (Supplementary Table S6). No statistical significance in PTE rates was observed among the three imaging modalities. MR-guided thermal ablation showed an RR of 1.06 (95%CI: 0.96–1.17, *p* = 0.90) compared to CT and 1.08 (95%CI: 0.98–1.20, *p* = 0.90) compared to US. The relative effects for different modality comparisons are shown in Figure 2B. According to the ranking analysis of the SUCRA score (Figure 3D), MR (SUCRA = 89.6) could be the best choice for improving primary technique effectiveness, but unfortunately, no statistical difference was achieved.

### 3.6. Major Complication

A total of six studies reported major complications based on SIR classification [37], but one study did not report the specific sample size and was, therefore, not included in the meta-analysis (Supplementary Table S6). No statistically significant differences in major complication rates were observed among the three imaging modalities. MR-guided thermal ablation demonstrated a relative risk (RR) of 0.27 (95% CI: 0.13–0.59, *p* = 0.94) compared to CT guidance, and 0.41 (95% CI: 0.10–1.74, *p* = 0.94) compared to US guidance. The relative effects for different modality comparisons are shown in Figure 3E. MR (SUCRA = 94.7) was ranked first again, followed by US (SUCRA = 42.2) and CT (SUCRA = 13.2). MR could be the guidance modality with the highest safety for thermal ablation.

**Figure 4:**
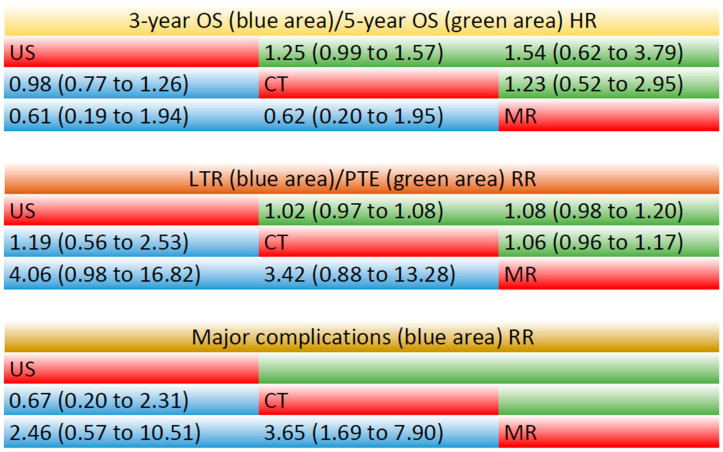
The league table shows hazard ratios (HRs) or relative ratios (RRs) for pairwise comparisons of the 3-year OS, 5-year OS, LTR, PTE, and major complications among the image modalities. The comparisons should be read from left to right. HRs (95%CI) or RRs (95%CI) for the comparisons are in the cells shared by the column-defining and row-defining interventions. CT, computed tomography; MR, magnetic resonance; US, ultrasound.

The relative effects for the different modality comparisons are provided in Figure 4. And, the pooled results of the network meta-analysis among CT, MR, and US are shown in Supplementary Figure S2.

### 3.7. Assessment of Inconsistency

There were no significant differences for 3-year OS, LTR, or PTE in terms of inconsistencies between the direct and indirect estimates in the node-splitting analysis within the closed loop in the evidence network (US-MR-CT) (Supplementary Table S7). The result of the 5-year OS and major complications could not be confirmed with an inconsistency analysis due to a lack of studies comparing MR and US (the triangle of the network was not complete).

## 4. Discussion

Over the past decade, advancements in percutaneous ablation techniques have significantly enhanced the treatment options for HCC patients, leading to improved local control and allowing more patients to benefit from these therapies [38,39]. Advancements in percutaneous ablation techniques are evident not only in the ablation methods themselves but also in the imaging modalities used to guide ablation. Since 1993, when Rossi et al. [40] from Italy first reported ultrasound-guided RFA for small HCC, and in 1994, when Seki et al. [41] from Japan reported ultrasound-guided MWA for small HCC, the use of image-guided thermal ablation for HCC has expanded significantly. This includes the increasingly widespread use of CT-guided thermal ablation [42] and the rapidly advancing MR-guided ablation techniques [43]. Although fusion imaging techniques such as US-CT [44] and US-MR [45] have been successfully applied in tumor thermal ablation, US, CT, and MR remain the most common and familiar imaging modalities used by clinicians to guide ablation. On the basis of this prevalence, we decided to publish the current network meta-analysis to offer a comprehensive and up-to-date overview of the available data in this field.

Thermal ablation is a type of energy-based ablation that targets specific tumor lesions within a particular organ, utilizing the biological effects generated by heat to directly induce irreversible damage or coagulative necrosis in tumor cells within the lesion tissue. The necrosis post-ablation encompasses not only the tumor itself but also the surrounding infiltrative capsule and adjacent areas in the liver that may harbor potential metastases [46]. Any energy-based ablation method should be considered thermal ablation, including RFA, MWA, cryoablation, and laser-induced thermotherapy (LITT). However, after a literature search, no imaging-guided comparisons were found for cryoablation and LITT. Therefore, this study only includes radiofrequency ablation and microwave ablation.

To our knowledge, this is the first systematic review and network meta-analysis that compares CT, MR, and US as imaging guidance modalities during thermal ablation therapy for HCC. With 2349 patients, this is also one of the largest meta-analyses conducted to date investigating image-guided thermal ablation modality for HCC patients. For 3-year and 5-year OS, although no significant differences were observed among the three imaging modalities across studies, rank probabilities indicated that US was ranked first for both 3-year OS (SUCRA = 68) and 5-year OS (SUCRA = 90). However, in the SUCRA rankings for both LTR and PTE, US was found to rank third (SUCRA = 17.4, 14.5, respectively), although this difference did not reach statistical significance. Despite the worst LTR and PTE in US, 3-year OS and 5-year OS were best after percutaneous ablation, even if not significantly. In order to understand these results, many factors should be considered: (1) The widespread availability and ease of operation of ultrasound equipment may contribute to its frequent clinical use. The extensive clinical application and experience gained from its usage could potentially result in higher overall survival rates, as more patients can receive effective treatment. (2) Moreover, patients undergoing ultrasound-guided thermal ablation may find it easier to undergo multiple follow-up visits and additional therapies, such as supplementary ablation, leading to improved overall survival rates.

Compared to the thermal ablation under CT guidance and US guidance, MR-guided thermal ablation was found to rank first in the SUCRA ranking for both LTR and PTE without statistically significant differences. The result may be explained on the basis of the advantages of MR, which has the ability to accurately evaluate the immediate efficacy after ablation [47,48]. During ultrasound-guided ablation, the tissue vaporization produced air bubbles, leading to a hyperechoic response in the ablation area, which obscured the original lesions. As a result, the precise extent of ablation could only be approximately estimated based on the hyperechoic region [49]. Similar to ultrasound-guided thermal ablation, CT-guided thermal ablation zones exhibited a combination of low-density changes, making it difficult to visualize a distinct boundary of the original lesion and accurately determine the safety margin [42,50]. In contrast to ultrasound and CT guidance, the post-ablation original lesion remained clearly visible on T1-weighted imaging (T1WI), appearing hypointense, surrounded by the hyperintense coagulation necrosis of liver tissue, exhibiting characteristic “target sign” changes with a distinct safety margin. Complete ablation was assessed when the lesion was entirely enveloped by the hyperintense ablation zone on T1WI, exceeding the safety margin of 5–10 mm [8,47,48]. Hence, the evaluation of complete ablation on MR imaging is more objective after the initial ablation, explaining why MR ranks first in SUCRA for both LTR and PTE.

Notably, while MRI offers precise immediate efficacy assessment and reduces local recurrence rates, it still faces significant challenges in routine clinical practice. MRI scanners are considerably more expensive than ultrasound machines, both in terms of initial investment and ongoing maintenance. Additionally, MRI-guided procedures require specialized MR-compatible ablation devices and dedicated facilities, further increasing the financial burden. Compared to ultrasound guidance, MRI-guided interventions also involve longer procedural times, adding logistical challenges for both healthcare providers and patients. The balance between practical potential cost and the clinical advantages of MRI should be carefully considered.

In this study, we focused on four key clinical outcomes—OS, LTR, PTE, and major complications. These endpoints were chosen as they directly reflect the effectiveness and safety of treatment, providing a comprehensive evaluation of clinical performance. Moreover, these clinical endpoints were the most commonly reported outcomes across all the included studies, ensuring consistency and comparability in our analysis. However, it is important to acknowledge that post-recurrence survival (PRS) is another critical outcome for evaluating the long-term efficacy of HCC treatments. Facciorusso et al. [51] found that local recurrences—defined as intrahepatic recurrences adjacent to the previously treated area—were associated with better PRS compared to distant intrahepatic recurrences or advanced-stage relapses, such as those with portal vein thrombosis or metastases. Unfortunately, as shown in Table 2, none of the studies included consistently reported PRS.

In our study, we also compared the safety of thermal ablation using these three imaging modalities. Among the 14 included studies, 6 studies classified complications based on the grading system of SIR. For major complications (classifications C-E), the rate of major complications in US-guided thermal ablation was higher than CT and MR, although not significantly. However, minor complications (classifications A-B), including fever, vomiting, pain, self-limiting intraperitoneal bleeding, etc., could not be evaluated as they were not uniformly reported in various studies.

This study is subject to several limitations. (1) As previously described, our network meta-analysis results primarily stem from non-randomized retrospective studies, which are susceptible to various biases and generally exhibit lower methodological rigor compared to RCTs. Specifically, the selection of imaging guidance modalities was primarily influenced by factors such as tumor visibility, location, operator experience, and equipment availability. Tumors in challenging anatomical locations, such as subdiaphragmatic or subcardiac regions, were more likely to be ablated with CT or MRI guidance due to superior imaging resolution and field of view. US-guided ablation was generally preferred for accessible tumors. However, none of the studies explicitly reported body habitus or tumor size as criteria for selecting a guidance method. Additionally, most of the studies were retrospective, and allocation bias cannot be completely ruled out. While some studies employed propensity score matching to reduce this bias, unmeasured confounding factors, such as operator preferences and institutional protocols, may still have influenced the allocation process. To address these issues, we conducted a comprehensive risk-of-bias assessment using the Newcastle–Ottawa Scale (NOS) and the ROBINS-I tool. Additionally, advanced statistical methods, including a frequentist approach for network meta-analysis and a random-effects model, were employed to mitigate the impact of heterogeneity and potential biases. Despite these efforts, the inherent limitations of retrospective studies may still influence the robustness of the conclusions. (2) There is a lack of direct comparison between MR and US; nevertheless, network meta-analysis enables the ranking of potential efficacy and safety profiles of various treatments, aiding in clinical decision making. (3) For OS, the majority of hazard ratios (HRs) and their SEs were derived from Kaplan–Meier curves, with potential inaccuracies arising from the imprecise plotting of points on the curves. (4) Due to the inconsistent stratification of tumor size in the included studies, we were unable to perform a subgroup analysis based on tumor size. (5) In our study, three of the included studies involved additional techniques: contrast enhancement [29], Lipiodol-assisted tumor localization [33], and intraoperative pneumothorax [27] to assist with puncture. These overlooked variables will inevitably affect the outcomes of ablation.

## 5. Conclusions

CT-, US-, and MR-guided thermal ablation are equally effective and safe for HCC patients.

## Figures and Tables

**Table 1:** Baseline characteristics of the study participants in the 14 included studies.

First Author	Year	Region	Study Design	Recruitment PERIOD	Ablation	Guidance-Modality	Sample Size	Age (mean ± SD)	%Male	Tumor Size (mean, cm)	Number of Tumors	NOS Score
Clasen, S [8]	2014	Germany	R	N.A.	RFA	CT	29	66.8 ± 9	86	3.49	29	7
						MR	24	64 ± 10.1		2.82	27	
Wu, J [24]	2015	China	R	2007–2012	RFA	CT	20	59.4 ± 10.3	75	N.A.	27	8
						US	20	52.3 ± 8.1	85	N.A.	24	
Lin, Z [25]	2016	China	R	2009–2014	RFA	CT	31	56.7	85	1.66	43	7
						MR	301				468	
						US	49				53	
Lee, L [26]	2017	Taiwan	R	2008–2013	RFA	CT	51	69 (median)	74.5	2.5 (median)	51	8
						US	101	71 (median)	63.4	2.5 (median)	101	
Hermida, M [27]	2018	France	R	2015–2017	RFA/MWA	CT	28	62.2 ± 9.2	89	1.5	28	8
						US	28	63.4 ± 11.2	79	1.7	28	
Huo, J [28]	2019	USA	R	2002–2011	RFA	CT	292	66 (at least)	61.4	N.A.	N.A.	6
						US	171		61.3			
Liu, Z [29]	2019	China	RCT	2013–2015	RFA	CT	56	54.5 ± 16.6	54	1.6	82	N.A.
						US	56	52.6 ± 13.7	57	1.5	88	
Yuan, C [30]	2019	China	R	2013–2016	RFA	CT	50	58.1 ± 10.4	86	1.6 (median)	N.A.	8
						MR	62	57.4 ± 7.5	89	2.0 (median)		
						US	29	57.4 ± 11.9	90	1.7 (median)		
Si, Z [31]	2020	China	R	2015–2017	RFA	CT	65	57 ± 10	70.8	2.2	N.A.	8
						US	68	58 ± 10	85.3	2.2		
Li, Z [32]	2021	China	R	N.A.	MWA	CT	47	55.8 ± 8.9	66	N.A.	N.A.	7
						MR	54	53.2 ± 6.5	80			
Wu, C [33]	2021	Taiwan	R	2016–2018	RFA	CT	184	66.3 ± 10.6	63.6	2.1	N.A.	8
						US	301	66.2 ± 10.6	64.5	2.2		
Yu, Z [34]	2021	China	R	N.A.	RFA	CT	47	50.9 ± 7.4	72.34	4.12	N.A.	8
						US	51	51.6 ± 7.2	80.39	4.11		
Mitani, H [35]	2022	Japan	R	2009–2016	RFA	CT	24	75 ± 7.6	79	1.23	30	7
						US	22	74 ± 7.3	68	1.11	26	
Zhao, W [36]	2022	China	R	2017–2019	MWA	CT-US	34	53 ± 10.8	71	3.4	88	8
						CT	30	50 ± 11.6	67	3.0		
						US	24	54 ± 11.4	67	3.2		

R, retrospective study; RCT, randomized controlled trial; CT, computed tomography; MR, magnetic resonance; US, ultrasound; RFA, radiofrequency ablation; MWA, microwave ablation; NOS, the Newcastle–Ottawa scale (NOS); N.A., not available.

**Table 2:** All clinical endpoints reported in the included studies.

	Study	Clasen, S. 2014 (CT vs. MR) [8]	Wu, J. 2015 (CT vs. US) [24]	Lin, Z. 2016 (CT vs. MR vs. US) [25]	Lee, L. 2017 (CT vs. US) [26]	Hermida, M. 2018 (CT vs. US) [27]	Huo, J. 2019 (CT vs. US) [28]	Liu, Z. 2019 (CT vs. US) [29]	Yuan, C. 2019 (CT vs. MR vs. US) [30]	Si, Z. 2020 (CT vs. US) [31]	Li, Z. 2021 (CT vs. MR) [32]	Wu, C. 2021 (CT vs. US) [33]	Yu, Z. 2021 (CT vs. US) [34]	Mitani, H. 2022 (CT vs. US) [35]	Zhao, W. 2022 (CT-US vs. CT vs. US) [36]
Endpoint															
Overall survival															
Local tumor progression															
Local tumor recurrence rate															
Local recurrence-free survival															
Progression-free survival															
Technical success rate															
Primary technique effectiveness rate															
Secondary technique effectiveness rate															
Major complication rate (SIR classification C-E)															
Minor complication rate (SIR classification A-B)															
Complication rate															
Adverse reaction rate															
Procedure time															
				Endpoint reporting							Endpoint non-reporting				

CT, computed tomography; MR, magnetic resonance; US, ultrasound; SIR, Society of Interventional Radiology classification.

## Data Availability

Data are contained within the article or Supplementary Material.

## Supplementary Materials

The following supporting information can be downloaded at https://www.mdpi.com/article/10.3390/cancers17030409/s1, Figure S1: The summary of the risk-of-bias assessment. **A.** Summary of bias risk assessment for retrospective studies **B.** Traffic light diagram of retrospective studies; Figure S2: The pooled results of the network meta-analysis among CT, MR, and US. **A.** The 3-year OS and 5-year OS. **B.** Local tumor recurrence, primary technique effectiveness, and major complications. HR, hazard ratio; RR, relative ratio; CI, confidence interval; CT, computed tomography; MR, magnetic resonance; US, ultrasound; Table S1: The detailed search strategy for all four databases incl. search terms used for the systematic literature searches; Table S2: List of excluded studies, with reasons for exclusion after full-text reading; Table S3: The Newcastle–Ottawa scale (NOS) quality assessment of the cohort studies included in the meta-analysis; Table S4: Confounding factors in retrospective studies, ROBINS-I tool for assessment of non-randomized interventional studies; Table S5: Overall survival at 3 years and 5 years in META-analysis; Table S6: Local tumor recurrence rate, primary technique effectiveness rate, and major complication rate in META-analysis; Table S7: Node-splitting analysis of inconsistency between direct and indirect effects in a closed loop network (US-MR-CT) in terms of 3-year overall survival (OS), local tumor recurrence (LTR), and primary technique effectiveness (PTE); Table S8: The PRISMA for Network Meta-Analysis Checklist.

## Abbreviations

HCC	hepatocellular carcinoma
MR	magnetic resonance
CT	computed tomography
US	ultrasound
HR	hazard ratio
RR	relative risk
CI	confidence interval
OS	overall survival
LTR	local tumor recurrence
PTE	primary technique effectiveness
RFA	radiofrequency ablation
MWA	microwave ablation
