# Supplementary material for: A Comparison of the Efficacy and Safety of US-, CT-, and MR-Guided Radiofrequency and Microwave Ablation for HCC: A Systematic Review and Network Meta-Analysis
Source: Cancers (Basel). 2025 Jan 26;17(3):409. doi: 10.3390/cancers17030409 (PMC11816381; doi:10.3390/cancers17030409)
Supplement: Supplementary file 1 [file cancers-17-00409-s001.zip › Figure S1.pdf]

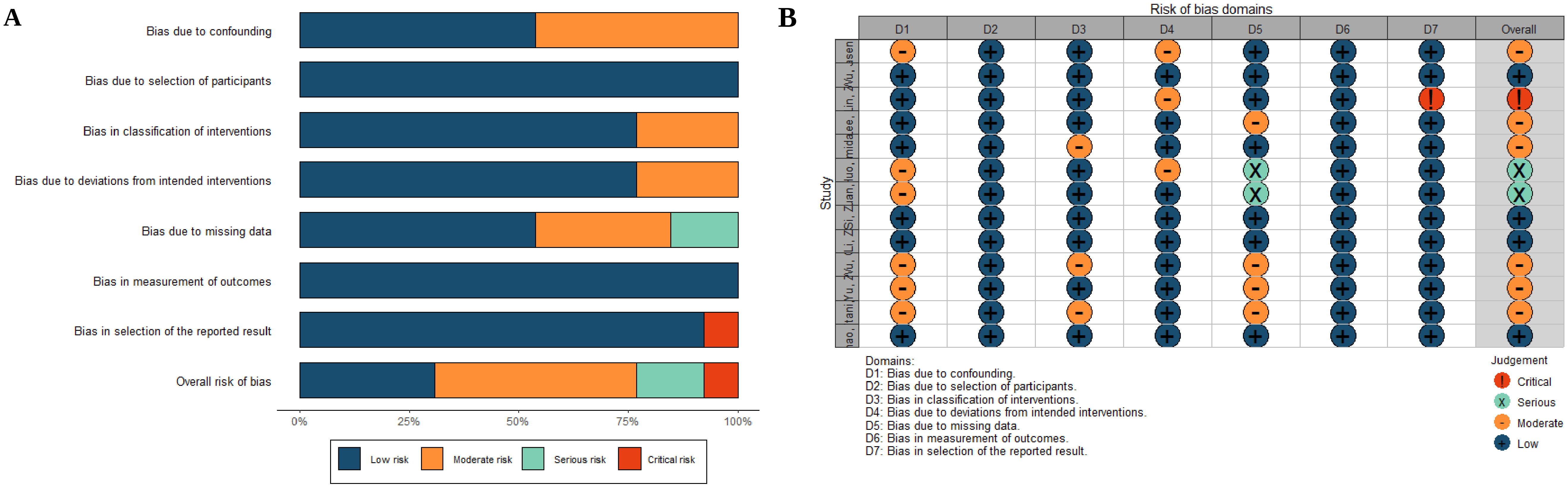

Supplementary Figure S1. The summary of the risk of bias assessment. **A.** Summary of bias risk assessment for retrospective studies **B.** Traffic light diagram of retrospective studies.
