# Supplementary material for: A Comparison of the Efficacy and Safety of US-, CT-, and MR-Guided Radiofrequency and Microwave Ablation for HCC: A Systematic Review and Network Meta-Analysis
Source: Cancers (Basel). 2025 Jan 26;17(3):409. doi: 10.3390/cancers17030409 (PMC11816381; doi:10.3390/cancers17030409)
Supplement: Supplementary file 1 [file cancers-17-00409-s001.zip › Figure S2.pdf]

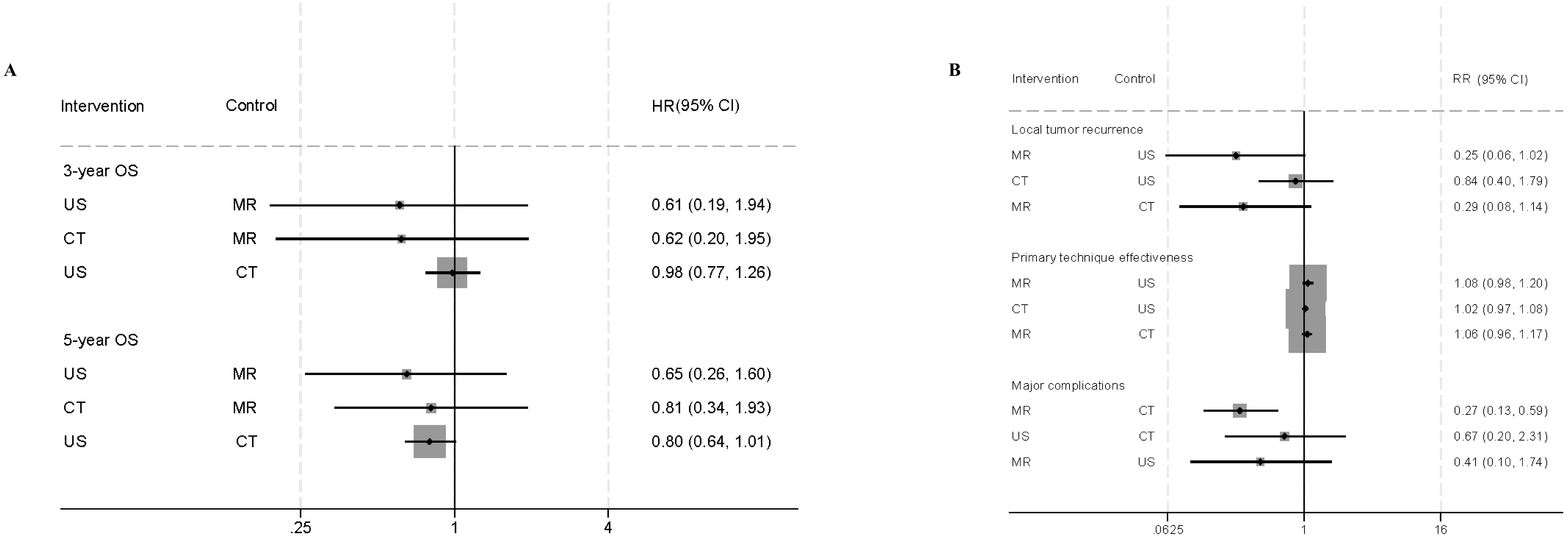

Supplementary Figure S2. The pooled results of the network meta-analysis among CT, MR and US. **A.** 3-year OS and 5-year OS. **B.** Local tumor recurrence, Primary technique effectiveness and Major complications. HR, hazard ratio; RR, relative ratio; CI, confidence interval; CT, Computed Tomography; MR, Magnetic Resonance; US, Ultrasound.
