# Supplementary material for: A Comparison of the Efficacy and Safety of US-, CT-, and MR-Guided Radiofrequency and Microwave Ablation for HCC: A Systematic Review and Network Meta-Analysis
Source: Cancers (Basel). 2025 Jan 26;17(3):409. doi: 10.3390/cancers17030409 (PMC11816381; doi:10.3390/cancers17030409)
Supplement: Supplementary file 1 [file cancers-17-00409-s001.zip › Table S1 The detailed search strategy for all four databases incl. search terms used for the systematic literature searches.pdf]

Table S1. The detailed search strategy for all four databases incl. search terms used for the systematic literature searches

Database	Search Term	Result
**PubMed**	#1: hepatocellular carcinoma [Title/Abstract] OR liver cancer [Title/Abstract] OR hepatoma [Title/Abstract] OR Liver Neoplasms [Title/Abstract] OR hepatic carcinoma [Title/Abstract] OR primary liver cancer [Title/Abstract] OR hepatic tumor [Title/Abstract] OR hepatic malignancy [Title/Abstract] OR hepatocarcinoma [Title/Abstract] OR liver metastases [Title/Abstract] OR liver metastasis [Title/Abstract] OR secondary liver cancer [Title/Abstract] OR hepatic secondary tumor [Title/Abstract]	198,424
	#2: microwave ablation [Title/Abstract] OR MWA [Title/Abstract] OR radiofrequency ablation [Title/Abstract] OR RFA [Title/Abstract] OR cryoablation [Title/Abstract] OR radiofrequency therapy [Title/Abstract] OR microwave therapy [Title/Abstract] OR cryotherapy [Title/Abstract] OR laser therapy [Title/Abstract] OR laser thermotherapy [Title/Abstract] OR Laser interstitial thermal therapy [Title/Abstract] OR LITT [Title/Abstract] OR laser ablation [Title/Abstract]	57,190
	#3: MR-guided [Title/Abstract] OR Magnetic resonance [Title/Abstract] OR Interventional Magnetic Resonance Imaging [Title/Abstract] OR CT-guided [Title/Abstract] OR computed tomography [Title/Abstract] OR computed tomography-guided [Title/Abstract] OR ultrasound [Title/Abstract] OR ultrasound-guided [Title/Abstract]	1,013,438
	#4: #1 AND #2 AND #3	1,486
**Web of Science**	#1: TI= (hepatocellular carcinoma OR liver cancer OR hepatoma OR Liver Neoplasms OR hepatic carcinoma OR primary liver cancer OR hepatic tumor OR hepatic malignancy OR hepatocarcinoma OR liver metastases OR liver metastasis OR secondary liver cancer OR hepatic secondary tumor) OR AB= (Hepatocellular carcinoma OR liver cancer OR hepatoma OR Liver Neoplasms OR hepatic carcinoma OR primary liver cancer OR hepatic tumor OR hepatic malignancy OR hepatocarcinoma OR liver metastases OR liver metastasis OR secondary liver cancer OR hepatic secondary tumor)	350,382
	#2: TI= (microwave ablation OR MWA OR radiofrequency ablation OR RFA OR cryoablation OR radiofrequency therapy OR microwave therapy OR cryotherapy OR laser therapy OR laser thermotherapy OR Laser interstitial thermal therapy OR LITT OR laser ablation) OR AB= (microwave ablation OR MWA OR radiofrequency ablation OR RFA OR cryoablation OR radiofrequency therapy OR microwave therapy OR cryotherapy OR laser therapy OR laser thermotherapy OR Laser interstitial thermal therapy OR LITT OR laser ablation)	154,075

	#3: TI= (MR-guided OR Magnetic resonance OR Interventional Magnetic Resonance Imaging OR CT-guided OR computed tomography OR computed tomography-guided OR ultrasound OR ultrasound-guided) OR AB= (MR-guided OR Magnetic resonance OR Interventional Magnetic Resonance Imaging OR CT-guided OR computed tomography OR computed tomography-guided OR ultrasound OR ultrasound-guided)	1,354,799
	#4: #1 AND #2 AND #3	2,175
**EMBASE**	#1: 'hepatocellular carcinoma':ab,ti OR 'liver cancer':ab,ti OR hepatoma:ab,ti OR 'liver neoplasms':ab,ti OR 'hepatic carcinoma':ab,ti OR 'primary liver cancer':ab,ti OR 'hepatic tumor':ab,ti OR 'hepatic malignancy':ab,ti OR hepatocarcinoma:ab,ti OR 'liver metastases':ab,ti OR 'liver metastasis':ab,ti OR 'secondary liver cancer':ab,ti OR 'hepatic secondary tumor':ab,ti	272,850
	#2: 'microwave ablation':ab,ti OR mwa:ab,ti OR 'radiofrequency ablation':ab,ti OR rfa:ab,ti OR cryoablation:ab,ti OR 'radiofrequency therapy':ab,ti OR 'microwave therapy':ab,ti OR cryotherapy:ab,ti OR 'laser therapy':ab,ti OR 'laser thermotherapy':ab,ti OR 'laser interstitial thermal therapy':ab,ti OR litt:ab,ti OR 'laser ablation':ab,ti	80,191
	#3: 'mr guided': ab,ti OR 'magnetic resonance':ab,ti OR 'interventional magnetic resonance imaging':ab,ti OR 'ct guided':ab,ti OR 'computed tomography':ab,ti OR 'computed tomography-guided':ab,ti OR ultrasound:ab,ti OR 'ultrasound guided':ab,ti	1,311,195
	#4: #1 AND #2 AND #3	2,116
**Cochrane Library**	#1: (hepatocellular carcinoma OR liver cancer OR hepatoma OR Liver Neoplasms OR hepatic carcinoma OR primary liver cancer OR hepatic tumor OR hepatic malignancy OR hepatocarcinoma OR liver metastases OR liver metastasis OR secondary liver cancer OR hepatic secondary tumor): ti	5,947
	#2: (hepatocellular carcinoma OR liver cancer OR hepatoma OR Liver Neoplasms OR hepatic carcinoma OR primary liver cancer OR hepatic tumor OR hepatic malignancy OR hepatocarcinoma OR liver metastases OR liver metastasis OR secondary liver cancer OR hepatic secondary tumor): ab	15,159
	#3: #1 OR #2	16,460
	#4: (microwave ablation OR MWA OR radiofrequency ablation OR RFA OR cryoablation OR radiofrequency therapy OR microwave therapy OR cryotherapy OR laser therapy OR laser thermotherapy OR Laser interstitial thermal therapy OR LITT OR laser ablation): ti	6,616

	#5: (microwave ablation OR MWA OR radiofrequency ablation OR RFA OR cryoablation OR radiofrequency therapy OR microwave therapy OR cryotherapy OR laser therapy OR laser thermotherapy OR Laser interstitial thermal therapy OR LITT OR laser ablation): ab	12,903
	#6: #4 OR #5	14,446
	#7: (MR-guided OR Magnetic resonance OR Interventional Magnetic Resonance Imaging OR CT-guided OR computed tomography OR computed tomography-guided OR ultrasound OR ultrasound-guided): ti	19,092
	#8: (MR-guided OR Magnetic resonance OR Interventional Magnetic Resonance Imaging OR CT-guided OR computed tomography OR computed tomography-guided OR ultrasound OR ultrasound-guided): ab	65,082
	#9: #7 OR #8	70,115
	#10: #3 AND #6 AND #9	146
**Time**	**31 May 2023**	
