# Supplementary material for: A Comparison of the Efficacy and Safety of US-, CT-, and MR-Guided Radiofrequency and Microwave Ablation for HCC: A Systematic Review and Network Meta-Analysis
Source: Cancers (Basel). 2025 Jan 26;17(3):409. doi: 10.3390/cancers17030409 (PMC11816381; doi:10.3390/cancers17030409)
Supplement: Supplementary file 1 [file cancers-17-00409-s001.zip › Table S2 List of excluded studies, with reasons for exclusion after full text reading.pdf]

Table S2. List of excluded studies, with specific reasons for exclusion after full text reading

Excluded reasons	First Author	Year	Region	Title	Specific reasons for exclusion
Reason 1: No comparison between imaging modalities	Orlacchio, A	2008	Italy	Percutaneous cryoablation of small hepatocellular carcinoma with US guidance and CT monitoring: initial experience	Cryoablation, monitored with computed tomography (CT) or ultrasonographic (US), for the treatment of hepatocellular carcinoma (HCC), but no comparison between these two image modalities;
	Fairchild, A.H	2014	USA	Percutaneous Cryoablation of Hepatic Tumours Adjacent to the Gallbladder: Assessment of Safety and Effectiveness	Cryoablation under computed tomography (CT) or magnetic resonance imaging (MRI) guidance, but no comparison between these two image modalities;
	Yang, Y	2020	China	Efficacy and Safety of Percutaneous Argon-Helium Cryoablation for Hepatocellular Carcinoma Abutting the Diaphragm	Percutaneous argon-helium cryoablation (CA), ultrasound (US) and computed tomography (CT) combined with US guidance, but no comparison between these two image modalities;

Reason 2:<br>Conference Abstract	Gao, F	2015	N.A.	Supradiaphragmatic computed tomography and infradiaphragmatic ultrasound guidance for microwave ablation of hepatic dome tumours	Conference Abstract;
	Adwan, H	2022	Germany	CT-guided microwave ablation versus MR-guided laser-induced thermotherapy of hepatocellular carcinoma	
	Liang, P	2022	China	Short-term safety, effectiveness, and influencing factors of microwave ablation in Chinese patients with hepatocellular carcinoma: a prospective multi-centre study	

Reason 3: No relevant outcomes	Wang, C	2012	China	Tumour seeding after percutaneous cryoablation for hepatocellular carcinoma	Cryoablation, guided by ultrasonography (US) and spiral computed tomography (CT) in treatment of HCC, but only analysed the risk factors for seeding, without pertinent results related to this study;
	Ge, Y	2012	China	The values of contrast-enhanced computed tomography and ultrasound in radiofrequency ablation for liver cancer	RFA, under computed tomography (CT) or ultrasound (US) guidance in treatment of liver tumours, only assessed the values of CEUS and CECT in the evaluation of therapeutic effect of RFA, without pertinent results related to this study;
Reason 4: In Japanese language	Yasumoto, T	2005	Japan	Radiofrequency ablation for hepatocellular carcinoma and liver metastases	Except for the abstract, the main body is written in Japanese;
