# Supplementary material for: A Comparison of the Efficacy and Safety of US-, CT-, and MR-Guided Radiofrequency and Microwave Ablation for HCC: A Systematic Review and Network Meta-Analysis
Source: Cancers (Basel). 2025 Jan 26;17(3):409. doi: 10.3390/cancers17030409 (PMC11816381; doi:10.3390/cancers17030409)
Supplement: Supplementary file 1 [file cancers-17-00409-s001.zip › Table S3 The Newcastle-Ottawa Scale (NOS) quality assessment of cohort studies included in the meta-analysis.pdf]

Table S3. The Newcastle-Ottawa Scale (NOS) quality assessment of retrospective studies included in the meta-analysis*

First author, publication year	Selection				Comparability	Outcome			Total score
	I	II	III	IV	V †	VI	VII ‡	VIII §	
Clasen, S. 2014	/	☆	☆	☆	☆	☆	☆	☆	7
Wu, J. 2015	/	☆	☆	☆	☆☆	☆	☆	☆	8
Lin, Z. 2016	/	☆	☆	☆	☆	☆	☆	☆	7
Lee, L. 2017	/	☆	☆	☆	☆☆	☆	☆	☆	8
Hermida, M. 2018	/	☆	☆	☆	☆☆	☆	☆	☆	8
Huo, J. 2019	☆	/	/	☆	☆	☆	☆	☆	6
Yuan, C. 2019	/	☆	☆	☆	☆☆	☆	☆	☆	8
Si, Z. 2020	/	☆	☆	☆	☆☆	☆	☆	☆	8
Li, Z. 2021	/	☆	☆	☆	☆☆	☆	☆	/	7
Wu, C. 2021	/	☆	☆	☆	☆☆	☆	☆	☆	8
Yu, Z. 2021	/	☆	☆	☆	☆☆	☆	☆	☆	8
Mitani, H. 2022	/	☆	☆	☆	☆☆	☆	☆	/	7
Zhao, W. 2022	/	☆	☆	☆	☆☆	☆	☆	☆	8

I, Representativeness of the exposed cohort; II, Selection of the non-exposed cohort; III, Ascertainment of exposure; IV, Demonstration that outcome of interest was not present at start of study; V, Comparability of cohorts on the basis of the design or analysis; VI, Assessment of outcome; VII, Was follow-up long enough for outcomes to occur; VIII, Adequacy of follow-up of cohorts.

* A study can be awarded a maximum of one star for each numbered item within the selection and outcome/exposure categories. However, a maximum of two stars can be given for an item in the comparability category.

† A maximum of 2 stars could be awarded for this item. Studies that controlled for tumor size (the most important factor) received one star; Study controls for tumor number, tumor location, Alpha-fetoprotein (AFP), BCLC stage, Albumin (Alb), Aspartate Aminotransferase (AST), Alanine Aminotransferase (ALT), hepatitis B virus, hepatitis C virus received one star.

‡ A cohort study with a follow-up time > 1 year was assigned one star.

§ A cohort study with a follow-up rate > 95%, or description provided of the lost was assigned one star.
