# Supplementary material for: A Comparison of the Efficacy and Safety of US-, CT-, and MR-Guided Radiofrequency and Microwave Ablation for HCC: A Systematic Review and Network Meta-Analysis
Source: Cancers (Basel). 2025 Jan 26;17(3):409. doi: 10.3390/cancers17030409 (PMC11816381; doi:10.3390/cancers17030409)
Supplement: Supplementary file 1 [file cancers-17-00409-s001.zip › Table S4 Confounding factors in retrospective studies, ROBINS-I tool for assessment of non-randomized interventional studies.pdf]

Table S4. Confounding factors in retrospective studies

Study	Guidance modality Arm	Tumor size(cm)			Ablation technique			Site of tumor				Follow-up protocol(months)	Patients lost to follow-up	Pre-TACE
		<3	>3	others	RFA	MWA	RFA and MWA	Peri-gallbladder	Peri-bowel	Subdiaphragmatic	Peri-vascular	CEUS or CECT or CEMR		
Clasen, S	CT	15	14	N.A.	Yes	No	No	N.A.	N.A.	N.A.	N.A.	1, 3 (12),6(>12)	N.A.	14/29
Clasen, S	MR	15	9	N.A.	Yes	No	No	N.A.	N.A.	N.A.	N.A.	1, 3 (12),6(>12)	N.A.	9/24
Wu, J	CT	15	9	N.A.	Yes	No	No	N.A.	N.A.	N.A.	N.A.	every 3-6	Yes	N.A.
Wu, J	US	17	10	N.A.	Yes	No	No	N.A.	N.A.	N.A.	N.A.		Yes	N.A.
Lin,Z	CT	43	0	N.A.	Yes	No	No	N.A.	N.A.	N.A.	7/43	1, every 2-3	N.A.	N.A.
Lin,Z	MR	468	0	N.A.	Yes	No	No	N.A.	N.A.	N.A.	77/468		N.A.	N.A.
Lin,Z	US	53	0	N.A.	Yes	No	No	N.A.	N.A.	N.A.	17/53		N.A.	N.A.
Lee, L	CT	N.A.	N.A.	<2 (23), >2(28)	Yes	No	No	0	2	19	12	every 3-4	Yes	N.A.
Lee, L	US	N.A.	N.A.	<2 (31), >2(70)	Yes	No	No	8	8	23	17		Yes	N.A.
Hermida, M	CT	N.A.	N.A.	<3.5 (28)	No	No	Yes	N.A.	N.A.	28	N.A.	3(24),6(>24)	No	27/28
Hermida, M	US	N.A.	N.A.	<3.5 (28)	No	No	Yes	N.A.	N.A.	28	N.A.		No	0/28
Huo, J	CT	88	N.A.	3-5(54), >5(41)	Yes	No	No	N.A.	N.A.	N.A.	N.A.	N.A.	N.A.	N.A.
Huo, J	US	57	N.A.	3-5(57), >5(19)	Yes	No	No	N.A.	N.A.	N.A.	N.A.		N.A.	N.A.
Yuan, C	CT	N.A.	N.A.	N.A.	Yes	No	No	N.A.	N.A.	N.A.	N.A.	1(3), 3(>3)	Yes	N.A.
Yuan, C	MR	N.A.	N.A.	N.A.	Yes	No	No	N.A.	N.A.	N.A.	N.A.		Yes	N.A.
Yuan, C	US	N.A.	N.A.	N.A.	Yes	No	No	N.A.	N.A.	N.A.	N.A.		Yes.	N.A.
Si, Z	CT	68	N.A.	N.A.	Yes	No	No	N.A.	N.A.	11	N.A.	1, 3(24), every 3-6(>24)	N.A.	N.A.
Si, Z	US	68	N.A.	N.A.	Yes	No	No	N.A.	N.A.	6	N.A.		N.A.	N.A.
Li, Z	CT	28	N.A.	3-5(19)	No	Yes	No	N.A.	N.A.	12	N.A.	1, every3	Yes	N.A.
Li, Z	MR	24	N.A.	3-5(30)	No	Yes	No	N.A.	N.A.	7	N.A.		Yes	N.A.
Wu, C	CT	N.A.	34	N.A.	Yes	No	No	N.A.	3	52	47	1-2, every 3, every 6	Yes	184/184
Wu, C	US	N.A.	34	N.A.	Yes	No	No	N.A.	7	73	57		Yes	0/301

Yu, Z	CT	N.A.	N.A.	N.A.	Yes	No	No	N.A.	N.A.	N.A.	N.A.	N.A.	Yes	N.A.
Yu, Z	US	N.A.	N.A.	N.A.	Yes	No	No	N.A.	N.A.	N.A.	N.A.		Yes	N.A.
Mitani, H	CT	30	N.A.	N.A.	Yes	No	No	N.A.	N.A.	30	5	7d, every 3-6	Yes	24/30
Mitani, H	US	26	N.A.	N.A.	Yes	No	No	N.A.	N.A.	26	5		Yes	22/26
Zhao, W	CT	N.A.	N.A.	N.A.	No	Yes	No	N.A.	N.A.	N.A.	N.A.	1,3,6,12	N.A.	N.A.
Zhao, W	US	N.A.	N.A.	N.A.	No	Yes	No	N.A.	N.A.	N.A.	N.A.		N.A.	N.A.

CT, Computed Tomography; MR, Magnetic Resonance; US, Ultrasound; RFA, Radiofrequency Ablation; MWA, Microwave Ablation; CE-US, Contrast enhanced ultrasound; CE-MR, Contrast enhanced magnetic resonance imaging; CE-CT, Contrast enhanced computed tomography; TACE, Transcatheter arterial chemoembolization; N.A., Not Available.
