# Supplementary material for: A Comparison of the Efficacy and Safety of US-, CT-, and MR-Guided Radiofrequency and Microwave Ablation for HCC: A Systematic Review and Network Meta-Analysis
Source: Cancers (Basel). 2025 Jan 26;17(3):409. doi: 10.3390/cancers17030409 (PMC11816381; doi:10.3390/cancers17030409)
Supplement: Supplementary file 1 [file cancers-17-00409-s001.zip › Table S5 Overall survival at 3 years and 5 years in META-Analysis.pdf]

Table S5. Overall survival at 3 years and 5 years in META-Analysis

	First Author	Year	Guidance-Modality	Procedure	HR (95% CI)
Overall survival at 3 years	Wu, J	2015	CT vs. US	RFA	1.33 (0.36-4.84) *
	Lee, L	2017	CT vs. US	RFA	1.25 (0.38-4.21)
	Hermida, M.	2018	CT vs. US	RFA/MWA	1.44 (0.21-10.03) *
	Huo, J	2019	CT vs. US	RFA	0.98 (0.74-1.30) *
	Liu, Z	2019	CT vs. US	RFA	2.58 (0.69-9.56) *
	Yuan, C	2019	CT vs. US	RFA	0.42 (0.04-4.96) *
			CT vs. MR		0.31 (0.07-1.40) *
			MR vs. US		0.76 (0.10-5.74) *
	Li, Z	2021	CT vs. MR	MWA	2.42 (0.24-24.76) *
	Wu, C	2021	CT vs. US	RFA	1.19 (0.30-4.73) *
	Yu, Z	2021	CT vs. US	RFA	0.63 (0.22-1.74) *
Overall survival at 5 years	Wu, J	2015	CT vs. US	RFA	1.26 (0.46-3.43) *
	Huo, J	2019	CT vs. US	RFA	1.22 (0.96-1.56)
	Li, Z	2021	CT vs. MR	MWA	0.81 (0.34-1.94)
	Wu, C	2021	CT vs. US	RFA	1.88 (0.63-5.60)

CT, Computed Tomography; MR, Magnetic Resonance; US, Ultrasound; RFA, Radiofrequency Ablation; MWA, Microwave Ablation; HR, Hazard Ratio; CI, Confidence Interval.

* Data extracted from the survival curve
