# Supplementary material for: A Comparison of the Efficacy and Safety of US-, CT-, and MR-Guided Radiofrequency and Microwave Ablation for HCC: A Systematic Review and Network Meta-Analysis
Source: Cancers (Basel). 2025 Jan 26;17(3):409. doi: 10.3390/cancers17030409 (PMC11816381; doi:10.3390/cancers17030409)
Supplement: Supplementary file 1 [file cancers-17-00409-s001.zip › Table S6 Local tumour recurrence rate, Primary technique effectiveness rate, and major complication rate in META-Analysis.pdf]

Table S6. Local tumour recurrence rate, Primary technique effectiveness rate, and major complication rate in META-Analysis

First Author	Year	Guidance-Modality	Procedure	Event/Sample size		
				Local tumour recurrence	Primary technique effectiveness	Major complication
Clasen, S	2014	CT	RFA	5/26	23/29	2/29
		MR		3/26	26/27	1/24
Wu, J	2015	CT	RFA	4/27	24/27	N.A.
		US		4/24	19/24	
Lin, Z	2016	CT	RFA	6/43	N.A.	N.A.
		MR		15/468		
		US		12/53		
Lee, L	2017	CT	RFA	12/51	47/51	0/51
		US		5/101	90/101	1/101
Hermida, M	2018	CT	RFA/MWA	3/28	28/28	N.A.
		US		7/28	28/28	
Liu, Z	2019	CT	RFA	12/56	41/56	N.A.
		US		4/56	46/56	
Yuan, C	2019	CT	RFA	N.A.	47/50	N.A.
		MR			60/62	
		US			27/29	
Si, Z	2020	CT	RFA	3/65	65/65	N.A.
		US		13/68	65/68	
Li, Z	2021	CT	MWA	N.A.	N.A.	21/47
		MR				6/54
Wu, C	2021	CT	RFA	N.A.	N.A.	2/160
		US				1/160
Yu, Z	2021	CT	RFA	2/47	29/47	N.A.
		US		3/51	18/51	
Zhao, W	2022	CT	MWA	2/30	N.A.	4/30
		US		4/24		2/24

CT, Computed Tomography; MR, Magnetic Resonance; US, Ultrasound; RFA, Radiofrequency Ablation; MWA, Microwave Ablation; N.A., Not Available.
