# Supplementary material for: A Comparison of the Efficacy and Safety of US-, CT-, and MR-Guided Radiofrequency and Microwave Ablation for HCC: A Systematic Review and Network Meta-Analysis
Source: Cancers (Basel). 2025 Jan 26;17(3):409. doi: 10.3390/cancers17030409 (PMC11816381; doi:10.3390/cancers17030409)
Supplement: Supplementary file 1 [file cancers-17-00409-s001.zip › Table S7 Node-splitting analysis of inconsistency between direct and indirect effects in a closed loop network (US-MR-CT) in terms of 3-year overall survival (O.pdf]

Table S7. Node-splitting analysis of inconsistency between direct and indirect effects in a closed loop network (US-MR-CT) in terms of 3-year overall survival (OS), local tumor recurrence (LTR) and primary technique effectiveness (PTE)

**Nodes**	**Direct effect**	**Indirect effect**	**Overall(network)**	***P*-value**
3-year OS				
MR, CT	1.76 (0.50 to 6.20)	1.05 (0.07 to 15.08)	1.60 (0.51 to 5.00)	0.73
US, CT	0.99 (0.77 to 1.26)	0.00 (0.00 to 17.18)	0.98 (0.77 to 1.26)	0.19
MR, US	1.35 (0.16 to 11.29)	1.76 (0.45 to 6.96)	1.63 (0.52 to 5.17)	0.84
LTR				
MR, CT	0.35 (0.08 to 1.54)	0.11 (0.00 to 3.35)	0.29 (0.08 to 1.14)	0.52
US, CT	1.13 (0.53 to 2.43)	9.65 (0.06 to 1651.56)	1.19 (0.56 to 2.53)	0.41
MR, US	0.14 (0.02 to 0.95)	0.50 (0.06 to 4.21)	0.25 (0.06 to 1.02)	0.39
PTE				
MR, CT	1.07 (0.96 to 1.19)	0.99 (0.78 to 1.27)	1.06 (0.96 to 1.17)	0.58
US, CT	0.97 (0.92 to 1.03)	1.51 (0.81 to 2.81)	0.98 (0.92 to 1.03)	0.17
MR, US	1.04 (0.90 to 1.20)	1.14 (0.98 to 1.33)	1.08 (0.98 to 1.20)	0.39
